# Factors Associated with Sleep Problems in Children with ADHD: Focusing on Emotional Regulation, Emotional Intensity and Internalizing Symptoms

**DOI:** 10.3390/bs16030404

**Published:** 2026-03-10

**Authors:** Doga Sevincok, Hasan Can Ozbay, Mutlu Muhammed Ozbek, Doruk Gul

**Affiliations:** 1 Department of Child and Adolescent Psychiatry, Istinye University, Istanbul 34396, Türkiye; 2 Department of Child and Adolescent Psychiatry, Tekirdag Ismail Fehmi Cumalioglu City Hospital, Tekirdag 59030, Türkiye; hasancan.ozbay@saglik.gov.tr; 3 Department of Child and Adolescent Psychiatry, Yalova University, Yalova 77200, Türkiye; mutlu.ozbek@yalova.edu.tr; 4 Department of Pediatrics, Istinye University, Istanbul 34396, Türkiye; doruk.gul@istinye.edu.tr

**Keywords:** ADHD, emotional intensity, emotion regulation, oppositional defiant disorder, internalization, sleep

## Abstract

The current study aimed to investigate sleep problems in children with Attention Deficit Hyperactivity Disorder (ADHD) within a framework highlighting emotion regulation (ER), emotional intensity (EI), oppositional defiant symptoms, and internalizing symptoms. A total of 100 children with ADHD and 50 controls aged 6–14 were recruited from University Hospital, and were assessed with semi-structured interviews. Parents completed the Children’s Sleep Habits Questionnaire, Conners’ Parent Rating Scale–Revised-Short, Emotion Regulation Scale for Children–Adult Form, and the Revised Children Anxiety and Depression Scale-Parent. Group comparisons, correlations, multiple regressions, and serial mediation models were conducted, adjusting for age, gender, and other covariates. After correction for multiple comparisons, sleep parameters and internalizing symptoms did not differ between groups. In the ADHD group, total sleep problems were correlated with ADHD and oppositional symptoms, EI, ER, and internalizing symptoms. Regression models indicated that internalizing symptoms predicted total sleep problems, while EI predicted night wakings. Across mediation models, internalizing symptoms consistently mediated associations between ADHD/oppositional symptoms and total sleep problems, with EI/ER contributing indirectly via internalization. Findings suggest that sleep problems related to ADHD are related to pathways of emotional distress, emphasizing the importance of assessing internalizing symptoms concurrently with behavioral/emotional processes during the evaluation of sleep problems.

## 1. Introduction

Attention Deficit Hyperactivity Disorder (ADHD) is among the most commonly diagnosed conditions in children and adolescents, with the diagnosis rates around 5–7% (Polanczyk et al., 2014) worldwide, and 12.4% in our country (Ercan et al., 2019). The core symptoms of ADHD include functionally impairing age-inappropriate attention deficit, hyperactivity, and impulsivity that begin before 12 years of age (APA, 2013). Despite the impairing effects of core symptoms of the disorder, some other co-occurring internalization or externalization problems, like depression, anxiety, oppositional defiant disorder, and conduct disorder, may also negatively affect children’s daily lives (Schatz & Rostain, 2006; Tung et al., 2016). As a prevalent problem in childhood and adolescence with ADHD, sleep problems have emerged as a substantial research area. It is believed that children with ADHD have many more sleep problems, such as bedtime resistance, sleep onset issues, sleep breathing problems, and parasomnias (Cortese et al., 2009). Evidence suggests that those sleep problems were more prevalent among children with ADHD, with high prevalences (Becker, 2020; Becker et al., 2019). There are also findings from objective measures that sleep problems in ADHD are more common than in other children (Liang et al., 2023). It has been suggested that sleep disorders may increase the severity of core symptoms of ADHD, further impair academic performance, and subsequently be related to several emotional and behavioral issues (Hvolby, 2015; Liang et al., 2024). Additionally, behavioral sleep problems are associated with a wide range of internalizing and externalizing symptoms in children with ADHD, leading to functional impairments in the child’s life (Becker et al., 2019; Dimakos et al., 2021).

The mechanisms underlying sleep problems in children with ADHD are considered to be multifactorial (Owens, 2005). Taking into account he correlation between ADHD and sleep problems, dysregulation of common arousal and attentional systems, and the possible negative effects of psychotropic medication used in ADHD, may be potential factors for this comorbidity (Corkum et al., 2007; Cortese et al., 2009; Mindell & Owens, 2010; Owens, 2005). However, the exact mechanisms between sleep and ADHD symptoms are yet to be investigated. One potential mechanism is internalizing symptoms, as well as some behavioral issues (Accardo et al., 2012). It has been proposed that sleep problems might be associated with anxiety and behavior problems (Mick et al., 2000). In a recent ADHD study, investigators found that internalizing symptoms were higher in poor sleepers, and the parameters were correlated with each other (Liang et al., 2024). Furthermore, Frick et al. (2023) postulated that emotional problems might be an important mediator between ADHD symptoms and sleep problems. Thus, it is reasonable to investigate further mechanisms for the ADHD–sleep relationship, considering the effects of internalizing symptoms and some other variables.

One possible mechanism for the explanation of sleep problems in ADHD children is emotional regulation (ER). ER is defined as the ability to identify, interpret, and control emotional expressions and to modulate the severity or duration of them, to maintain control in social situations with socially accepted responses in an adaptive manner (Esbjørn et al., 2012; Gratz & Roemer, 2004; Paulus et al., 2021; Thompson, 1994). Emotional dysregulation (ED), which refers to difficulties in ER, is characterized by hyperarousal, irritability, anger, excessive sadness, or fear (Tonacci et al., 2019). Problems in ER have been found to be associated with internalizing and externalizing conditions, as well as difficulties in emotional development, self-efficacy, social relationships, and quality of life (Biederman et al., 2012; Dugal et al., 2018; Loevaas et al., 2018; Paulus et al., 2021; Tonacci et al., 2019). Research indicates that ADHD is associated with substantial ER problems, including high emotional instability, increased negative affect, and difficulties in expressing emotions (Bunford et al., 2018; Huguet et al., 2019; Lugo-Candelas et al., 2017; Overgaard et al., 2018; Özbaran et al., 2018). ER difficulties might be considered a core feature of ADHD, even in the absence of a comorbid diagnosis (Soler-Gutiérrez et al., 2023).

In addition to ADHD symptoms, oppositional defiant symptoms have also been associated with ER problems, such as temper tantrums, fears, and low tolerance to frustration (Déry et al., 2017; Steiner & Remsing, 2007). Those oppositional defiant symptoms may also be associated with depression (Déry et al., 2017). Importantly, a longitudinal study found that sleep problems in early childhood were associated with ED, and both were persistent over time in children with ADHD and typically developing children (Williams & Sciberras, 2016). Sleep problems and these ER difficulties appear to develop in an interconnected manner over time, with the development of one elevating the likelihood of the other emerging (Wang et al., 2019).

Taken together, it is reasonable to investigate ADHD, oppositional defiant symptoms, internalizing symptoms, and ER difficulties as potential factors associated with sleep problems. While previous research has shown a bidirectional association between sleep problems and emotional distress, our study is based on a developmental model. Emotional intensity (EI) and ER are conceptualized as proximate emotional processes closely linked to ADHD symptomatology. High EI and ER difficulties are considered substantial features of ADHD and are thought to increase vulnerability to internalizing symptoms, such as anxiety and depression. Internalizing symptoms, in turn, represent a substantial psychological correlation with childhood sleep problems. Therefore, internalizing symptoms are placed after emotional processes, and sleep problems are treated as the outcome variable to investigate whether ADHD-related EI and ER difficulties are associated with sleep problems through internalizing symptoms.

In this study, our first aim was to assess group differences in sleep parameters between ADHD and typically developing children. We also aimed to investigate group differences for internalizing symptoms, ER, and EI. We also hypothesized that not only psychological symptoms but also some problems with electronic exposure (mobile phone usage before sleep and total screen time) may be more common in ADHD children. In this study, our second aim was to explore the relationships between various sleep problems and ER, EI, and other clinical symptoms in children with ADHD and in typically developing children, and to assess differences in correlation patterns between groups. Given that a large number of factors may influence sleep problems, and considering the interrelationships among these factors, analyses were conducted while controlling for other relevant variables. Therefore, regression analyses were appropriate for this purpose. Lastly, we aimed to evaluate factors that may account for variance in the relationships between ADHD symptoms and sleep parameters, and between behavioral symptoms and sleep difficulties. Therefore, examination of factors such as ER, EI, and internalizing symptoms in their mediating roles was of significant importance.

## 2. Materials and Methods

### 2.1. Participants

Our study was conducted with children and adolescents aged 6–14 and their parents, who were recruited at Istinye University Hospital. The sample size of our study was calculated using the G*Power program version 3.1 (Dusseldorf, Germany), based on the study by Owens et al. (2000a), with a type 1 error of 0.05 and a power of 0.90. A total of 100 children and adolescents were included in the ADHD group, and 50 in the control group. Cases in the ADHD group were recruited from the child and adolescent psychiatry outpatient clinic from patients who had been evaluated for possible ADHD diagnosis or were followed up with in our hospital routinely. Inclusion criteria for the case group were as follows: (1) clinical diagnosis of ADHD according to DSM-5 (The Diagnostic and Statistical Manual of Mental Disorders-Fifth Edition) criteria following a semi-structured clinical evaluation; (2) non-existence of any medical condition that might affect sleep or influence scale scores; (3) normal intellectual capacity, clinically consistent with normal cognitive functioning, both in the children and in their parents, who fulfill the scales; and (4) willingness of the child and their parents to participate in the study. Participants were excluded from the study if they had major psychological conditions like schizophrenia, bipolar disorder, autism spectrum disorder, intellectual disability, or substance abuse. They were also excluded if more than 10% of the items in each scale were missing. Control group children were recruited from the outpatient pediatric clinic at the same hospital from patients who had been admitted for routine medical evaluation. Inclusion criteria for the control group were as follows: (1) non-existence of any psychiatric disorder history and not having any diagnosis for a psychological condition; (2) non-existence of any medical condition that might affect sleep or influence scale scores; (3) normal intellectual capacity, clinically consistent with normal cognitive functioning, both in the children and in their parents, who fulfill the scales; and (4) willingness of the child and their parents to participate in the study. For the control group, participants with a previous or current psychiatric diagnosis identified through clinical evaluation, or with a history of psychotropic medication use, were excluded from the study. This study was conducted in accordance with the Declaration of Helsinki, and written informed consent was obtained from the parents of all participants after a detailed explanation of the study procedures. Ethical approval for the study was obtained from the Ethics Committee of Istinye University (Ref. No.: 2025-260).

### 2.2. Procedure

All the possible ADHD diagnosed children and controls were assessed with a semi-structured interview by an experienced child and adolescent psychiatrist (D.S.). This semi-structured interview took place with both children and their parents. Afterwards, patients who had a confirmed ADHD diagnosis were included as the case group, while children who had no psychiatric diagnosis and were admitted to the pediatric outpatient clinic were included as the control group. After the inclusion and exclusion criteria were met, parents were instructed to complete the scales based on their children’s recent behavioral patterns. Participants could take breaks whenever they felt tired or bored and started again once they felt rested. The clinician (D.S.) was blinded to the questionnaire scores.

### 2.3. Instruments

#### 2.3.1. Sociodemographic Data Form

This form was prepared by the researchers in order to determine the sociodemographic and developmental properties of the study and the control groups. In this form, some variables, such as age, gender, family structure, mobile phone use before sleep, total screen time, and ADHD medication, were assessed.

#### 2.3.2. Kiddie Schedule for Affective Disorders and Schizophrenia (K-SADS-PL)

K-SADS-PL is a semi-structured interview used to assess psychiatric disorders in children and adolescents based on DSM-5 criteria (Kaufman et al., 2016). Unal et al. (2019) adapted the Turkish version and tested its reliability and validity. The tool has three main parts. The first part is an unstructured interview that looks at developmental history, medical condition, and general functioning. The second part uses screening questions to find current and past psychiatric symptoms. The last part helps identify clinically important symptom areas based on the screening. In this study, K-SADS-PL was used to confirm ADHD diagnoses, check for psychiatric comorbidities, and decide eligibility according to inclusion and exclusion criteria.

#### 2.3.3. Children’s Sleep Habits Questionnaire (CSHQ)

The Children’s Sleep Habits Questionnaire (CSHQ), developed by Owens et al. (2000b) to assess children’s sleep habits and sleep-related problems, comprises 33 items. The instrument includes eight subscales: bedtime resistance (items 1, 3, 4, 5, 6, 8), delayed onset of sleep (item 2), sleep duration (items 9, 10, 11), sleep anxiety (items 5, 7, 8, 21), nocturnal awakenings (items 16, 24, 25), parasomnias (items 12, 13, 14, 15, 17, 22, 23), sleep-disordered breathing (items 18, 19, 20), and daytime sleepiness (items 26, 27, 28, 29, 30, 31, 32, 33). The questionnaire is completed retrospectively by parents, who are instructed to evaluate their child’s sleep habits over the previous weeks. Items are scored as follows: generally (behavior occurs 5–7 times per week): 3; sometimes (2–4 times per week): 2; and rarely (0–1 time per week): 1. Items 1, 2, 3, 10, 11, and 26 are reverse-coded (generally: 1; sometimes: 2; rarely: 3). Items 32 and 33 are coded as follows: does not feel sleepy: 0; very sleepy: 1; falls asleep: 2. The Turkish validity and reliability study of the scale was conducted by Perdahlı Fiş et al. (2010) in school-aged children. In our study, the Cronbach’s alpha value for this scale was 0.804, indicating very good internal consistency.

#### 2.3.4. Conners’ Parent Rating Scale-Revised: Short Form (CPRS-R-S)

This scale was created by Conners (1997) by selecting the items with the highest factor loadings as a result of exploratory factor analysis applied to data collected for revised long forms. The CPRS-R-S consists of 27 items. The items are grouped into three subscales (oppositional defiant disorder, cognitive problems/inattention, and hyperactivity) and one auxiliary scale (ADHD index). A high score indicates that the child has the problems defined in the CPRS-R-S more often. The Turkish validity and reliability study of the scale was conducted by Kaner et al. (2013). In our study, the Cronbach’s alpha value for this scale was 0.961, indicating excellent internal consistency.

#### 2.3.5. Emotion Regulation Scale for Children-Adult Form (ERSC-A)

This scale, developed by Rydell et al. (2003), assesses children’s emotional intensity (EI) and emotion regulation (ER) abilities as two distinct constructs. These constructs are evaluated across four domains: anger, fear, exuberance or excitement, and sadness. In each domain, four of the ten items measure emotional intensity, while the remaining six assess emotion regulation, yielding a total of 40 items. The scale uses a 5-point Likert format, ranging from 1 to 5, and may be completed by a parent, caregiver, or teacher. Higher scores on the EI subscale indicate higher levels of emotional reactivity and increased emotional difficulties. In contrast, higher scores on the ER subscale reflect more advanced and adaptive ER skills. The item “Has difficulty calming himself/herself” in each domain is reverse-scored. The scale is available in a short version with 16 items and a long version with 40 items, and the long version was utilized in the present study. The adaptation study of its Turkish form was conducted by Tatlı Harmancı and Güngör Aytar (2023). In our study, Cronbach’s alpha values for this scale were 0.898 for EI and 0.910 for ER, demonstrating very good and excellent internal consistency, respectively.

#### 2.3.6. Revised Child Anxiety and Depression Scale-Parent Form: (RCADS-P)

The RCADS-P is a 47-item questionnaire used to assess DSM-IV depression and anxiety disorders in children and adolescents (Chorpita et al., 2000). It uses a 4-point Likert scale, where 0 means never, 1 means sometimes, 2 means often, and 3 means always. This scale provides six subscales, an Anxiety Total Score (the sum of the five anxiety scales), and a Total (Internalizing) Score (the sum of all six subscales). Gormez et al. (2017) studied the Turkish version and found it had good psychometric properties. In our study, we used only the total internalization score, and the Cronbach’s alpha for the total scale was 0.930.

### 2.4. Data Analysis

All analyses were performed with the Statistical Package for the Social Sciences (SPSS), version 22 (IBM Corporation, Armonk, NY, USA). Initially, all variables were examined for normality assumptions using skewness and kurtosis values and histogram graphs, and potential outliers were addressed appropriately. If fewer than 10% of the values were missing, they were replaced with the item’s mean score. Although the mean assignment method can reduce variance, the low proportion of missing data at the item level suggests that the potential effect will be minimal.

We used G*Power to perform a power analysis before the study to determine the required sample size. Considering the normal distribution of our data in each group, parametric tests were preferred. For group comparisons, Student’s T test was performed for continuous variables, and means and standard deviations were reported. Bonferroni correction was applied to adjust the significance threshold for multiple comparisons. This correction method applied to primary hypothesis-driven comparisons to control family-wise error. For categorical variables, the Chi-square test was conducted, reporting number (*n*) and percentage (%) values. Furthermore, Pearson’s correlation analyses were used to examine associations between sleep parameters and other study variables, separately in the study and control groups, and Pearson correlation coefficients (r) were reported. For all correlation analyses, False Discovery Rate (FDR) correction was applied to control for multiple comparisons while maintaining statistical power. The rationale for conducting different correction methods (FDR) in these exploratory secondary analyses involving multiple correlated outcomes was to balance Type 1 error and statistical power and to reduce excessive Type 2 error, considering the high conservative nature of the Bonferroni correction. Additionally, multiple linear regression analyses were performed to test the associations of dependent variables (CSHQ subscales) with associated factors, after controlling for other variables and age. Therefore, with the “enter method”, nine regression models with five independent variables were tested for possible independent relationships. No additional correction for multiple testing was applied to the regression analyses, as each model examined a predefined outcome. A *p*-value of less than 0.05 was considered statistically significant in all analyses.

To investigate the direct and indirect effects of ADHD and oppositional defiant symptoms on sleep problems, four separate serial mediation models were built, and EI, ER, and internalizing symptoms were proposed as potential mediators. Age, gender, and ADHD treatment were included as covariates in the models in order to control their confounding effects. Direct and indirect effects were analyzed using the PROCESS V4.2 macro model for SPSS (Hayes, 2013). This program uses a bootstrapping method to estimate parameters. In our study, unstandardized coefficients (b) and 95% confidence intervals (95% CI) were reported. This technique is considered appropriate for smaller sample sizes and was applied in this study with 5000 bootstrap resamples (MacKinnon et al., 2004). Considering our variables, Model 6 was suitable to test the serial mediation, and CIs that do not include zero were accepted as a statistically significant indirect effect (Preacher & Hayes, 2008).

To mitigate potential item overlap between the RCADS and the sleep measure, the internalizing score was recalculated as additional analysis with the RCADS item explicitly assessing sleep problems (“I have trouble sleeping”) excluded. All correlation, regression, and serial mediation analyses were subsequently repeated using this revised internalizing score and given as Supplementary Materials.

Additionally, to assess the potential influence of phenotypic heterogeneity, ADHD presentation (combined/hyperactive versus predominantly inattentive) was included as a covariate in all multivariable and mediation models in Supplementary Materials. Due to the small number of participants with predominantly hyperactive–impulsive presentation (*n* = 4), this subgroup was merged with the combined presentation group to ensure statistical stability. Subgroup comparisons between ADHD presentations were conducted at the univariate level as additional analysis. For transparency and to maintain clarity in the primary analyses, the full results of these confirmatory and sensitivity models are provided in the Supplementary Material (Tables S1–S4).

## 3. Results

Our study sample consisted of 100 children diagnosed with ADHD and 50 healthy controls. A total of 80% of the ADHD children were boys, whereas this percentage was 76% in the control group. There were no statistically significant differences between groups in terms of gender (*p* = 0.673) and age (*p* = 0.870). Considering the ADHD presentation, most of the children were diagnosed as combined presentation (63%), and 33% of them were predominantly inattentive. Unexpectedly, total screen time (*p* = 0.529) and mobile phone time before sleep (*p* = 0.803) did not show any significant differences between groups. Additionally, despite some group differences in parasomnias and total sleep problems, group comparisons yielded no statistically significant differences for any sleep parameters, after Bonferroni correction. Furthermore, although the RCADS-Internalization scores were higher in ADHD children, this difference lost statistical significance after Bonferroni correction. As expected, EI was significantly lower (*p* = 0.001), and ER was considerably higher (*p* < 0.001) in the ADHD group; both remained significant after correction. Moreover, all CPRS-RS subscales were significantly higher in the ADHD group, which was expected. Overall, the key group-level finding was that EI and ER differed significantly between groups after Bonferroni correction, while sleep parameters and internalizing symptoms remained statistically insignificant. All descriptives and group comparisons are shown in Table 1.

According to our hypothesis, correlation analyses demonstrated significant relationships between sleep parameters and other variables in the study group, after FDR correction. Investigating total sleep problems, CSHG-Total was significantly associated with oppositional defiance (r = 0.468), ADHD index (r = 0.377), EI (r = 0.455), ER (r = −0.305), and RCADS-Internalization scores (r = 0.538), after FDR correction. There were also substantial relationships between the CSHG subscales and other variables, as shown in Table 2. Additionally, as shown in Table 3, some associations were also present in the control group. After FDR correction, CSHQ-Total was only related to RCADS-Internalization (r = 0.465), while CSHQ-SD was associated with mobile phone time before sleep (r = 0.460). Furthermore, internalizing symptoms were associated with both CSHQ-NW (r = 0.336) and CSHQ-P (r = 0.409), while emotional intensity was correlated only with CSHQ-P (r = 0.458). There were no statistically significant correlations for BR, SOD, SA, SDB, and DS in the control group. The key correlational finding in the ADHD group was that internalizing symptoms showed the strongest and most consistent association with overall sleep problems.

In this study, we conducted multiple linear regression analyses to determine the unique associations of each sleep problem domain with behavioral and emotional variables, while controlling for other variables in the ADHD group. Age, ADHD index, oppositional defiance symptoms, EI, ER, and internalizing symptoms were entered into all regression models as independent variables. In these regression analyses, the maximum VIF value was 2.516, indicating no multicollinearity. The results of the multiple regression analyses are shown in Table 4. In the model in which total sleep problems were included as the dependent variable, the overall model was statistically significant (F = 8.956, *p* < 0.001), explaining 36.6% of the variance in total sleep problems. After controlling age and other variables, only RCADS-Internalization remained significant in predicting total sleep problems (β = 0.443, *p* < 0.001, 95% CI [−1.20, 0.09]). In the models in which bedtime resistance and sleep anxiety were included as dependent variables separately, age (β = −0.288, *p* = 0.003, 95% CI [−0.68, −0.14]; β = −0.288, *p* = 0.003, 95% CI [−0.58, −0.22]; respectively) and RCADS-Internalization (β = −0.393, *p* < 0.001, 95% CI [0.02, 0.11]; β = 0.457, *p* < 0.001, 95% CI [0.02, 0.08]; respectively) variables were significant predictors in both models. In these models, the overall models were significant (F = 4.959, F = 7.749, respectively), explaining a substantial amount of variance. A different pattern emerged when the night wakings variable was considered as the dependent variable. In this model, the model was statistically significant (F = 5.325, *p* < 0.001), explaining 25.6% of the variance. EI was found to be a significant predictor of night wakings (β = 0.346, *p* = 0.017, 95% CI [0.01, 0.06]), along with age (β = −0.295, *p* = 0.002, 95% CI [−0.27, −0.06]), after controlling for other variables. The only predictor of sleep breathing, after controlling for age and other variables, was RCADS-Internalization (β = 0.354, *p* = 0.011, 95% CI [0.01, 0.04]). Regarding sleep onset delay, sleep duration, parasomnias, and daytime sleepiness, our regression models were insignificant, indicating no significant predictors among those we investigated in this study.

**Figure 1:**
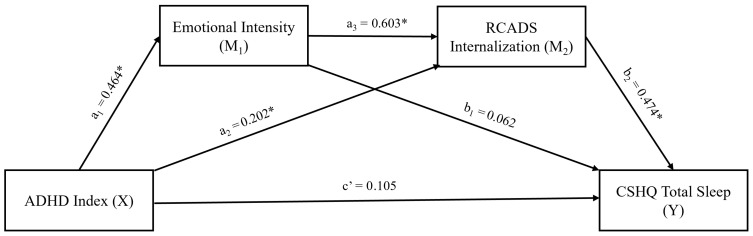
Results of serial mediation analysis, testing the mediator effects of emotional intensity and internalizing symptoms on the path from ADHD index to total sleep problems. * Statistically significant (*p* < 0.05 or CIs do not include zero). ADHD: Attention Deficit Hyperactivity Disorder, RCADS: Revised Child Anxiety and Depression Scale, CSHQ: Child Sleep Habits Questionnaire.

To investigate the direct and indirect effects of ADHD symptoms or oppositional defiant symptoms on sleep problems, we conducted four separate mediation models. In all the mediation models, age, gender, and ADHD treatment were included as covariates to control their potential confounding effects on sleep problems. Across all mediation models, RCADS-Internalization emerged as the important variable linking ADHD-related and oppositional symptoms to total sleep problems. In all the models, the total effect and the total indirect effect on total sleep problems (Y) were statistically significant, indicating substantial mediator effects of the examined variables. In the first mediation model (Table 5, Figure 1), we included ADHD index (X) as the independent variable. EI (M_1_) and RCADS-Internalization (M_2_) were included as serial mediators. The direct effect of ADHD index on EI (β = 0.464) and RCADS-Internalization (β = 0.202) was significant. The direct effect of EI on RCADS-Internalization was also significant (β = 0.603). Moreover, the direct effect of RCADS-Internalization on sleep problems was also significant (β = 0.474). However, the direct effect of ADHD index on sleep problems (β = 0.105) lost its significance after the inclusion of mediators. The indirect effect of ADHD index on sleep problems was statistically significant through internalizing symptoms (β = 0.095, 95% CI (0.030, 0.177)), as well as via serial mediation (β = 0.132, 95% CI (0.061, 0.220)). These findings were consistent with the mediator role of internalizing symptoms alone and of EI within the serial mediation. However, EI was not a unique mediator when internalizing symptoms were ignored.

**Figure 2:**
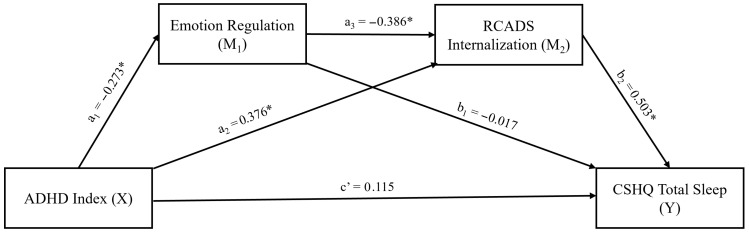
Results of serial mediation analysis, testing the mediator effects of emotion regulation and internalizing symptoms on the path from ADHD index to total sleep problems. * Statistically significant (*p* < 0.05 or CIs do not include zero) ADHD: Attention Deficit Hyperactivity Disorder, RCADS: Revised Child Anxiety and Depression Scale, CSHQ: Child Sleep Habits Questionnaire.

In the second mediation model (Table 6, Figure 2), ADHD index (X) was included as an independent variable, while ER (M_1_) and RCADS-Internalization (M_2_) were serial mediators. The direct effect of ADHD index on ER (β = −0.273) and RCADS-Internalization (β = 0.376) was significant. Additionally, the direct effect of RCADS-Internalization on sleep problems was also significant (β = 0.503). There was also a significant direct effect among mediator variables (β = −0.386). However, the direct effect of ADHD index on sleep problems (β = 0.115) was not significant, suggesting possible full mediation. In this second model, similar to the first model, the indirect effect of ADHD index on sleep problems was statistically significant through internalizing symptoms (β = 0.189, 95% CI (0.098, 0.294)), as well as via serial mediation (β = 0.053, 95% CI (0.009, 0.108)). These findings confirmed the possible mediator role of internalizing symptoms alone and of ER within the serial mediation. However, ER was not a unique mediator when internalizing symptoms were ignored.

**Figure 3:**
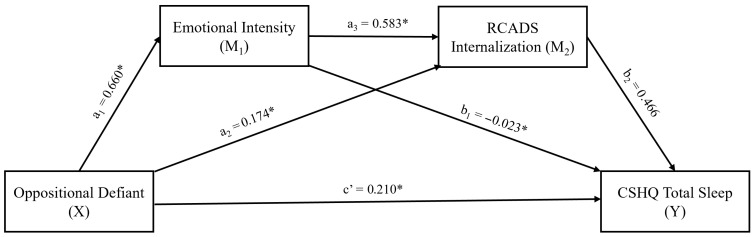
Results of serial mediation analysis, testing the mediator effects of emotional intensity and internalizing symptoms on the path from oppositional defiant symptoms to total sleep problems. * Statistically significant (*p* < 0.05 or CIs do not include zero). RCADS: Revised Child Anxiety and Depression Scale, CSHQ: Child Sleep Habits Questionnaire.

In the third mediation model (Table 7, Figure 3), oppositional defiant (X) symptoms were included as the independent variable, while EI (M_1_) and RCADS-Internalization (M_2_) were included as serial mediators. The direct effect of oppositional defiant symptoms on EI (β = 0.660) was significant. Additionally, the direct effect of RCADS-Internalization on sleep problems was also substantial (β = 0.466). There was also a significant direct effect among mediator variables (β = 0.583). However, the direct effect of oppositional defiant symptoms on RCADS-Internalization (β = 0.174) or total sleep problems (β = 0.210) was not significant. In this model, the indirect effect of oppositional defiant symptoms on sleep problems was significant only when EI and RCADS-Internalization were considered as serial mediators (β = 0.179, 95% CI (0.094, 0.286)). These findings confirmed the possible mediator role of ER and internalizing symptoms, only within the serial mediation.

**Figure 4:**
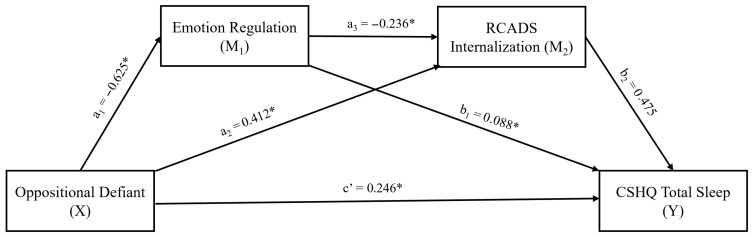
Results of serial mediation analysis, testing the mediator effects of emotion regulation and internalizing symptoms on the path from oppositional defiant symptoms to total sleep problems. * Statistically significant (*p* < 0.05 or CIs do not include zero). RCADS: Revised Child Anxiety and Depression Scale, CSHQ: Child Sleep Habits Questionnaire.

In our last mediation model (Table 8, Figure 4), ER (M_1_) was included as the first mediator instead of EI, while oppositional defiant symptoms were the independent variable (X). A similar pattern occurred, confirming the model’s stability. The direct effect of oppositional defiant symptoms on ER (β = −0.625), RCADS-Internalization (β = 0.412), and sleep problems (β = −0.625) was significant. Therefore, partial mediation was supported in this model. Furthermore, the direct effect of RCADS-Internalization on sleep problems was significant (β = 0.475). There was also a substantial direct effect among mediator variables (β = −0.236). In this last model, similar to the first two models, the indirect effect of ADHD index on sleep problems was statistically significant through internalizing symptoms (β = 0.195, 95% CI (0.074, 0.330)), as well as via serial mediation (β = 0.070, 95% CI (0.005, 0.159)). These findings supported the possible mediator role of internalizing symptoms alone and of ER within the serial mediation. However, ER was not a unique mediator when internalizing symptoms were ignored.

To determine whether item overlap influenced the association between internalizing symptoms and sleep problems, all analyses were repeated with the sleep-related item excluded from the RCADS-Internalization score. The correlation between internalizing symptoms and total sleep problems remained similar to the original model (r = 0.520 vs. r = 0.538). Internalizing symptoms continued to be independently associated with total sleep problems in multivariable regression analyses. Serial mediation analyses using the revised internalizing score produced a comparable overall pattern of results. The total indirect effects remained statistically significant across models, and the indirect pathway via internalizing symptoms was maintained (Supplementary Tables S1–S4).

To assess the potential impact of ADHD presentation (combined versus predominantly inattentive), additional analyses were conducted with ADHD presentation included as a covariate. Although children with combined presentation exhibited higher total sleep scores at the univariate level, ADHD presentation did not independently predict sleep problems in multivariable or mediation models. The primary indirect effects remained stable after adjustment for ADHD presentation (Supplementary Tables S2–S4).

Overall, these sensitivity analyses did not substantially alter the magnitude or interpretation of the primary findings.

## 4. Discussion

The present study explored sleep problems among children with ADHD through a comprehensive framework encompassing ER, EI, oppositional defiant symptoms, and internalizing symptoms. By making use of group comparisons, correlational analysis, regression analysis, and serial mediation analyses, this study has been pursued with the aim of not only understanding whether sleep difficulties among children with ADHD differ from their normally developing counterparts but also seeking to understand by what means emotional and behavioral constructs interact to impact sleeping difficulties. In this study, internalizing symptoms were found to be the most robust and consistent variable related to sleep problems among children with ADHD. Contrary to the large amount of literature that has supported the presence of heightened levels of sleep problems in children with ADHD (Becker, 2020; Cortese et al., 2009; Marten et al., 2023), the comparison of groups in the current analysis failed to find any statistically significant differences in most aspects of sleep after the correction for multiple comparisons. However, this result was consistent with work that has shown the absence of differences in subjective levels of sleep quality being experienced by ADHD versus non-ADHD groups, particularly when stringent control methods were applied (Dolapoglu et al., 2025; Loram et al., 2023). There could be many reasons for this trend. First, the issue of sleep in ADHD is highly heterogeneous. This varies with children’s age, comorbidities, medications used for co-occurring conditions, and parental attitudes (Becker et al., 2019; Marten et al., 2023). Secondly, the sleep data collected from parents may be less sensitive to detecting the group differences. This may be particularly true if behavioral disturbances have led to heightened reports of sleep problems (Fulfs et al., 2024). It is also important to note that the findings reflected perceived sleep difficulties rather than objective sleep problems. Additionally, the lack of group differences might be due to the nature of the control group. The control group was recruited from the outpatient pediatric clinics. This may have increased the likelihood of subclinical sleep or emotional complaints in the comparison group, potentially reducing observable between-group differences. Finally, because our sample included children between 6 and 14 years old, differences in sleep patterns related to age during this stage of development may have affected our results.

Despite no differences in sleep, there were some important differences between the two groups in emotional processes. ADHD children demonstrated a statistically significant increase in EI, meaning a predisposition to experience emotions with increased intensity and reactivity, along with a statistically significant decrease in ER, pointing to a decreased ability to control emotions in a flexible and adaptive manner. This pattern is highly consistent with the modern theories of emotional dysregulation, according to which dysregulation is defined not only by a deficit in regulatory control but also by the simultaneous presence of increased emotional intensity and decreased regulatory control (Bunford et al., 2018; Paulus et al., 2021). In line with this theory, it has been demonstrated that children suffering from ADHD tend to be characterized by rapid escalation of negative affect, increased emotional reactivity, and difficulty returning to a normal level of arousal following conditions of increased cognitive and interpersonal stress (Lugo-Candelas et al., 2017; Soler-Gutiérrez et al., 2023). Additionally, Paulus et al. (2021) emphasize that increased emotional intensity is a source of increased demand on regulatory systems, meaning that a relatively low level of impairment in ER is sufficient for the occurrence of considerable emotional dysregulation. In this respect, the combination of increased EI and low ER in our study is a developmentally and practically important pattern, demonstrated to be a considerable risk factor for a wide variety of psychopathology, including both internalizing and externalizing disorders (Bunford et al., 2018; Tonacci et al., 2019). Therefore, the inability to efficiently downregulate intense emotions may be associated with a considerable level of arousal, addressing a basic and important association between emotion regulation difficulties and further psychopathologies, which may lead to children suffering from sleep problems.

In line with previous studies, sleep problems in children with ADHD were shown to be significantly related to the severity of ADHD symptoms, symptoms of oppositional defiant disorder, EI, ER, and, more strongly, internalizing symptoms. The strong correlation between the overall sleep disturbance problems and the internalizing symptoms corroborates previous clinical and epidemiological research, where very close relationships between sleep problems and anxiety–depressive symptoms in children and adolescents were reported (Dong et al., 2024; Fulfs et al., 2024; Loram et al., 2023). Internalizing symptoms may be associated with sleep problems in a variety of ways, for instance, via cognitive hyperarousal, worries, rumination, or high levels of physiological arousal prior to sleep (Costa-López et al., 2023; Loram et al., 2023). On the other hand, poor sleep can also lead to an increase in emotional distress, thus offering evidence that is supportive of a reciprocal relationship that has also been replicated in previous studies on both ADHD and non-ADHD individuals (Becker et al., 2015; Loram et al., 2023; Wang et al., 2019). It is also important to note that in the control group, factors that influenced poor sleep were mostly related to internalizing symptoms and some factors related to the use of the mobile phone prior to bedtime, offering evidence that has been supported by previous literature that indicated that emotional distress might be a transdiagnostic correlate of poor sleep (Fulfs et al., 2024). In our study, although internalizing symptoms were significantly correlated with parasomnias, this relationship lost its significance in the regression model. Among parasomnias, non-REM parasomnias—such as sleepwalking and night terrors—are particularly relevant in pediatric populations and have been reported at higher rates in children with ADHD (Cortese et al., 2009, Tomic et al., 2025). Internalizing symptoms might also be an important factor in relation to non-REM parasomnias (Tomic et al., 2025). The lack of a strong association in our regression model may reflect a lack of subtype-specific assessment of non-REM parasomnias. Future studies should include a detailed assessment of parasomnia subtypes to clarify their independent contributions to internalization symptomatology in ADHD.

This study also evaluated the impact of phenotypic heterogeneity within ADHD, specifically combined versus predominantly inattentive presentations. Although children with the combined presentation showed higher levels of sleep difficulties in univariate analyses, ADHD presentation did not independently predict sleep outcomes in multivariable or mediation models. The primary indirect effects remained consistent after adjusting for presentation, showing that the mechanisms identified here apply across different ADHD presentations. Together, these supplementary analyses strengthen confidence in the findings and support the conclusion that internalizing symptoms play an important role in the association between ADHD symptom severity and sleep problems.

In this study, regression analyses conducted in children with ADHD helped clarify the relationship between emotional and behavioral variables with sleep problems in children with ADHD. Internalizing symptoms were identified as the primary factor in association with total sleep problems when considering age, severity of ADHD symptoms, oppositional defiant symptoms, EI, and ER in the model. This finding is consistent with previous literature indicating the importance of emotional symptoms for explaining variance in sleep problems beyond traditional symptoms of ADHD (Dong et al., 2024; Loram et al., 2023). Furthermore, the age of the children emerged as an important predictor of bedtime resistance and sleep anxiety, in which younger children face more challenges. In terms of development, for instance, younger children may express less mature self-regulatory skills in comparison to their older counterparts, hence being more prone to sleep problems due to anxiety issues (Fulfs et al., 2024). They are also more dependent on caregivers for regulating negative emotions, especially during transitions such as bedtime. The above data supports the developmental theories of sleep in children, which propose the presence of bedtime resistance and sleep anxiety as prominent in younger children but decreasing with increased maturity of regulatory autonomy and coping skills.

EI emerged as an important predictor of night-waking episodes in the ADHD group, even when accounting for the effects of age and other variables of emotions and behavior. This finding implies that high emotional reactivity may be linked to sleep arousal and disturbances. This has been observed in research linking emotional reactivity to parasomnia (Costa-López et al., 2023; Fulfs et al., 2024). The lack of predictors for sleep onset delay, sleep duration, parasomnia, and sleepiness in our study may be due to the complex and diverse nature of the determinants of these sleep parameters. Contrary to bedtime resistance and sleep anxiety, which are more closely associated with emotional and relationship aspects, the aforementioned parameters are more closely associated with the biological and environmental aspects. Previous research has shown that the presence of circadian preference, regularity of sleep habits, parental sleep quality, and family sleep habits are significant predictors of sleep onset timing, total sleep time, and sleepiness among children with ADHD (Bondopandhyay et al., 2024; Marten et al., 2023). Since these variables were not directly measured using the current models, the presence of emotional and behavioral symptoms may not have been sufficient to predict those sleep parameters.

A distinctive aspect of the current study is the use of serial mediation analyses. In all the models, the internalizing symptoms were the variable that mediated the link between ADHD/oppositional defiant symptoms and total sleep problems consistently. EI and ER were associated with sleep problems indirectly through their relationship with internalizing symptoms. This finding is consistent with theoretical models suggesting that difficulties in emotional regulation predict the emergence of symptoms related to anxiety and depression, which subsequently affect sleep (Costa-López et al., 2023; Frick et al., 2023; Loram et al., 2023). It is important to note that the two models (EI and ER) did not emerge as independent mediators when internalizing symptoms were excluded, indicating that emotional dysregulation alone may be insufficient to impair sleep unless it culminates in clinically relevant emotional distress.

Regarding ADHD symptoms, the findings supported full mediation, suggesting that the relationship between the severity of ADHD symptoms and sleep problems is mainly via emotional channels. On the other hand, for oppositional defiant symptoms, there was evidence of partial mediation in certain models, pointing to the existence of direct as well as indirect associations with sleep problems. Oppositional defiant symptoms could directly affect sleep by increasing bedtime resistance, coercive parenting, and increased physiological activity during bedtime. At the same time, persistent behavioral conflict and irritability could lead to increased emotional distress and internalizing problems, which, in turn, could affect sleep problems (Shanahan et al., 2014). This combination of results suggests that whereas the relationship between severity of ADHD symptoms and sleep problems is primarily mediated by emotional processes, the relationship between severity of oppositional defiant symptoms and sleep problems is consistent with the possibility of both direct and indirect associations.

Such results add to the existing body of research by demonstrating the substantial role of internalizing symptoms in the ADHD–sleep relationship. Although some studies have found links between sleep difficulties and emotional/behavioral issues (Becker et al., 2019; Dong et al., 2024), few studies have simultaneously examined the roles of EI, ER, and internalizing symptoms. This is consistent with meta-analytic evidence that subjective sleep problems in ADHD are more closely linked to emotional functioning than to objective sleep measures (Loram et al., 2023; Marten et al., 2023). Additionally, the current data resonates with the latest models that have conceptualized emotional distress as the central correlate in the pathogenesis between the occurrence of neurodevelopmental disorders and sleep problems (Costa-López et al., 2023).

There are some considerable limitations of this study. Firstly, the cross-sectional design of this current study naturally restricted us from making definite judgments about the causal direction of the variables. Accordingly, one should be cautious in interpreting these findings as evidence of causal relationships or definitive mechanistic pathways among ADHD symptoms, emotional processes, and sleep problems. It is important that longitudinal studies be used in the future to determine causal relationships. Secondly, there is an assumption that parent-report data may be vulnerable to the effects of shared method variance in the current investigation. In the future, multiple objective sources of data on sleep should be used. Thirdly, we did not use any measures for parental sleep and emotional functioning, which might influence factors associated with sleep in children with ADHD. Additionally, our study did not include NREM parasomnias, which have been reported to increase in children with ADHD. Therefore, subtype-specific associations could not be examined. Finally, the control group was recruited from a pediatric outpatient clinic rather than from the community. Therefore, those children could also have some subclinical sleep and emotional difficulties due to their mild medical conditions, which could reduce the likelihood of detecting significant differences for sleep parameters between the groups.

## 5. Conclusions

The present study demonstrates that sleep problems in children with ADHD are best understood within an emotional–psychopathological framework. Internalizing symptoms emerged as the noticeable clinical correlate linking ADHD and oppositional symptoms to sleep difficulties, while EI and ER contributed indirectly. These findings underscore the importance of integrative assessment and intervention strategies that address emotional functioning alongside sleep and behavioral concerns in children with ADHD. These findings suggest that it is important for the clinicians to explore internalizing symptomatology, even if anxiety or depression is not the chief complaint. Treatment approaches aimed at alleviating emotional distress, such as addressing anxiety with cognitive–behavioral approaches or emotion-based therapy, may have beneficial effects on sleep problems. Further, because of the possible mediating role of ER and EI, interventions that improve adaptive emotion regulation might decrease internalizing problems and, subsequently, sleep problems.

## Figures and Tables

**Table 1:** Comparison of sociodemographic and clinical variables between ADHD and control groups.

Variables	ADHD (*n* = 100) Mean ± SD	Control (*n* = 50) Mean ± SD	Statistical Analysis		
			t	*p*	Effect Size
**Age**	9.88 ± 2.30	9.95 ± 2.27	−0.164	0.870	d = 0.030
**Mobile Phone Time**	49.22 ± 51.60	47.00 ± 50.44	0.250	0.803	d = 0.043
**Total Screen Time**	198.30 ± 122.17	186.60 ± 98.71	0.631	0.529	d = 0.105
**CPRS-RS**					
CP/inattention	11.42 ± 4.38	4.46 ± 3.81	9.551	<0.001 *	d = 1.695
Hyperactivity	8.08 ± 4.69	2.60 ± 2.63	9.148	<0.001 *	d = 1.441
Oppositional defiance	9.99 ± 4.28	4.48 ± 3.21	7.240	<0.001 *	d = 1.456
ADHD index	22.10 ± 7.27	9.34 ± 6.19	10.630	<0.001 *	d = 1.889
**CSHQ**					
Bedtime resistance	9.63 ± 3.33	8.76 ± 3.07	1.545	0.125	d = 0.271
Sleep onset delay	1.56 ± 0.75	1.38 ± 0.63	1.533	0.128	d = 0.259
Sleep duration	3.84 ± 1.79	3.86 ± 1.41	−0.083	0.934	d = 0.012
Sleep anxiety	6.46 ± 2.36	6.34 ± 2.31	0.295	0.768	d = 0.051
Night wakings	4.17 ± 1.33	4.04 ± 1.04	0.602	0.548	d = 0.108
Parasomnias	9.16 ± 1.95	8.26 ± 1.46	2.870	0.005	d = 0.522
Sleep breathing	3.61 ± 1.15	3.32 ± 0.62	2.000	0.047	d = 0.313
Daytime sleepiness	12.86 ± 3.76	11.82 ± 3.42	1.644	0.102	d = 0.289
Total	47.85 ± 8.70	44.54 ± 7.43	2.301	0.023	d = 0.409
**ERSC-A**					
Emotional intensity	51.34 ± 12.89	43.88 ± 10.78	3.520	0.001 *	d = 0.627
Emotion regulation	82.33 ± 17.26	93.36 ± 11.58	−4.634	<0.001 *	d = 0.750
**RCADS**					
Internalization	29.99 ± 18.93	23.34 ± 12.28	2.589	0.011	d = 0.416
	***n* (%)**	***n* (%)**	**x^2^**	***p***	
**Gender**			0.318	0.673	
Boy	80 (80.0)	38 (76.0)			
Girl	20 (20.0)	12 (24.0)			
**Family Structure**			0.531	0.586	
Together	90 (90.0)	43 (86.0)			
Separated/divorced	10 (10.0)	7 (14.0)			
**ADHD Presentation**		-	-	-	
Inattention	33 (33.0)				
Hyperactivity	4 (4.0)				
Combined	63 (63.0)				
**ADHD Medication**		-	-	-	
Yes	61 (61.0)				
No	39 (39.0)				

* Bonferroni correction was applied; values that remain significant are flagged (threshold: *p* < 0.00238 = 0.05/21). CPRS-RS: Conners Parent Rating Scale-Revised Short; CSHQ: Children’s Sleep Habits Questionnaire; ERSC-A: Emotion Regulation Scale for Children-Adult Form; RCADS: Revised Children’s Anxiety and Depression Scale.

**Table 2:** Correlations between sleep parameters and other variables in ADHD children (*n* = 100).

Variables	CSHQ-BR	CSHQ-SOD	CSHQ- SD	CSHQ- SA	CSHQ- NW	CSHQ- P	CSHQ- SDB	CSHQ- DS	CSHQ- Total
**Age**	−0.260 *	0.127	0.089	−0.365 *	−0.331 *	−0.089	−0.106	0.115	−0.147
**Mobile phone time**	0.047	0.180	0.127	−0.018	−0.012	0.003	0.065	0.144	0.119
**Total screen time**	0.009	0.189	0.206	−0.037	−0.098	0.058	0.131	0.166	0.136
**CPRS-RS** Oppositional defiance	0.314 *	0.114	0.079	0.310 *	0.222	0.372 *	0.242 *	0.304 *	0.468 *
**CPRS-RS** ADHD index	0.250 *	0.088	0.037	0.206	0.230 *	0.317 *	0.203 *	0.247 *	0.377 *
**ERSC-A** Emotional intensity	0.288 *	−0.001	−0.008	0.351 *	0.387 *	0.302 *	0.249 *	0.315 *	0.455 *
**ERSC-A** Emotion regulation	−0.252 *	−0.038	−0.164	−0.252 *	−0.092	−0.214	−0.062	−0.178	−0.305 *
**RCADS**-Internalization	0.386 *	0.140	0.155	0.404 *	0.270 *	0.351 *	0.328 *	0.316 *	0.538 *

Note. Pearson correlation coefficients are reported. False discovery rate (Benjamini–Hochberg) correction was applied within this table. Correlations marked with an asterisk * are significant after FDR correction. CPRS-RS: Conners Parent Rating Scale-Revised Short; CSHQ: Children’s Sleep Habits Questionnaire; BR: bedtime resistance; SOD: sleep onset delay; SD: sleep duration; SA: sleep anxiety; NW: night wakings; P: parasomnias; SDB: sleep disordered breathing; DS: daytime sleepiness; ERSC-A: Emotion Regulation Scale for Children-Adult Form; RCADS: Revised Children’s Anxiety and Depression Scale.

**Table 3:** Correlations between sleep parameters and other variables in the control group (*n* = 50).

Variables	CSHQ- BR	CSHQ- SOD	CSHQ- SD	CSHQ- SA	CSHQ- NW	CSHQ- P	CSHQ- SDB	CSHQ- DS	CSHQ- Total
**Age**	−0.290	−0.036	0.254	−0.321	−0.315	−0.273	−0.140	0.216	−0.113
**Mobile phone time**	0.022	0.215	0.460 *	−0.053	0.211	0.250	0.194	0.293	0.353
**Total screen time**	0.121	0.164	0.235	0.021	0.122	0.072	0.135	0.291	0.305
**CPRS-RS** Oppositional defiance	0.119	0.269	−0.088	0.013	0.073	0.211	0.095	0.112	0.183
**CPRS-RS** ADHD index	0.041	0.210	0.103	−0.095	0.189	0.172	0.067	0.172	0.195
**ERSC-A** Emotional intensity	0.246	0.230	−0.195	0.288	0.183	0.458 *	0.103	−0.045	0.256
**ERSC-A** Emotion regulation	−0.097	−0.108	0.079	−0.027	−0.146	−0.037	−0.096	−0.208	−0.189
**RCADS**-Internalization	0.221	0.056	0.118	0.336	0.336 *	0.409 *	0.103	0.283	0.465 *

Note. Pearson correlation coefficients are reported. False discovery rate (Benjamini–Hochberg) correction was applied within this table. Correlations marked with an asterisk * are significant after FDR correction. CPRS-RS: Conners Parent Rating Scale-Revised Short; CSHQ: Children’s Sleep Habits Questionnaire; BR: bedtime resistance; SOD: sleep onset delay; SD: sleep duration; SA: sleep anxiety; NW: night wakings; P: parasomnias; SDB: sleep disordered breathing; DS: daytime sleepiness; ERSC-A: Emotion Regulation Scale for Children-Adult Form; RCADS: Revised Children’s Anxiety and Depression Scale.

**Table 4:** Results of multiple linear regression analyses conducted to investigate variables associated with sleep parameters in ADHD children (*n* = 100).

Variables	β	*p*	Variables	β	*p*
DV: bedtime resistance ^a^			DV: parasomnias ^f^		
Age	−0.288	0.003 *	Age	−0.081	0.409
ADHD index	0.021	0.859	ADHD index	0.081	0.514
Oppositional defiance	0.131	0.391	Oppositional defiance	0.256	0.106
EI	−0.137	0.344	EI	−0.050	0.736
ER	−0.031	0.803	ER	0.064	0.616
RCADS-Internalization	0.398	0.003 *	RCADS-Internalization	0.249	0.064
R^2^ = 0.242, F = 4.959	*p* < 0.001		R^2^ = 0.187, F = 3.576	*p* = 0.003	
DV: sleep onset delay ^b^			DV: sleep breathing ^g^		
Age	0.096	0.360	Age	−0.121	0.225
ADHD index	−0.010	0.939	ADHD index	−0.033	0.793
Oppositional defiance	0.211	0.214	Oppositional defiance	0.206	0.199
EI	−0.227	0.159	EI	0.005	0.976
ER	0.057	0.678	ER	0.236	0.071
RCADS-Internalization	0.210	0.145	RCADS-Internalization	0.354	0.011 *
R^2^ = 0.064, F = 1.063	*p* = 0.390		R^2^ = 0.159, F = 2.938	*p* = 0.011	
DV: sleep duration ^c^			DV: daytime sleepiness ^h^		
Age	0.049	0.640	Age	0.153	0.127
ADHD index	−0.013	0.919	ADHD index	0.033	0.794
Oppositional defiance	0.042	0.804	Oppositional defiance	0.172	0.285
EI	−0.266	0.099	EI	0.168	0.270
ER	−0.192	0.162	ER	0.084	0.518
RCADS-Internalization	0.211	0.142	RCADS-Internalization	0.135	0.321
R^2^ = 0.072, F = 1.197	*p* = 0.315		R^2^ = 0.159, F = 2.937	*p* = 0.011	
DV: sleep anxiety ^d^			DV: CSHQ-Total ^i^		
Age	−0.393	<0.001 *	Age	−0.147	0.093
ADHD index	−0.073	0.516	ADHD index	0.030	0.784
Oppositional defiance	0.106	0.459	Oppositional defiance	0.248	0.076
EI	−0.043	0.752	EI	0.004	0.977
ER	0.016	0.890	ER	0.081	0.470
RCADS-Internalization	0.457	<0.001 *	RCADS-Internalization	0.443	<0.001 *
R^2^ = 0.333, F = 7.749	*p* < 0.001		R^2^ = 0.366, F = 8.956	*p* < 0.001	
DV: night wakings ^e^					
Age	−0.295	0.002 *			
ADHD index	0.062	0.603			
Oppositional defiance	−0.046	0.758			
EI	0.346	0.017 *			
ER	0.174	0.156			
RCADS-Internalization	0.137	0.288			
R^2^ = 0.256, F = 5.325	*p* < 0.001				

Dubin–Watson = 1.661 ^a^, 2.087 ^b^, 2.122 ^c^, 1.710 ^d^, 2.072 ^e^, 1.858 ^f^, 2.076 ^g^, 1.734 ^h^, 2.044 ^i^. Note. Standardized beta values are reported. *p* values marked with an asterisk * are statistically significant. DV: dependent variable; EI: emotional intensity; ER: emotion regulation; RCADS: Revised Children’s Anxiety and Depression Scale.

**Table 5:** Results of serial mediation analysis, testing the mediator effects of emotional intensity and internalizing symptoms on the path from ADHD index to total sleep problems (*n* = 100).

Path	Standardized Coefficients	S.E.	*p*	95% CI	
				Lower	Upper
**Direct Effects**					
X→M_1_ (a_1_)	0.464 *	0.158	<0.001	0.508	1.137
X→M_2_ (a_2_)	0.202 *	0.212	0.014	0.107	0.952
M_1_→M_2_ (a_3_)	0.603 *	0.121	<0.001	0.650	1.133
M_1_→Y (b_1_)	0.062	0.080	0.603	−0.118	0.202
M_2_→Y (b_2_)	0.474 *	0.054	<0.001	0.108	0.325
X→Y (c’)	0.105	0.116	0.282	−0.105	0.356
**Indirect Effect**					
X→M_1_→Y	0.028	0.049	-	−0.061	0.137
X→M_2_→Y	0.095 *	0.037	-	0.030	0.177
X→M_1_→M_2_→Y	0.132 *	0.040	-	0.061	0.220
**Total Effect (c)**	0.362 *	0.112	<0.001	0.210	0.657

Note. Age, sex and ADHD treatment were included as covariates. * Statistically significant (*p* < 0.05 or CIs do not include zero). S.E.: standard error; X = ADHD index; M_1_ = emotional intensity; M_2_ = Revised Children’s Anxiety and Depression Scale-Total Internalization; Y = Children’s Sleep Habits Questionnaire-Total Sleep.

**Table 6:** Results of serial mediation analysis, testing the mediator effects of emotion regulation and internalizing symptoms on the path from ADHD index to total sleep problems (*n* = 100).

Path	Standardized Coefficients	S.E.	*p*	95% CI	
				Lower	Upper
**Direct Effects**					
X→M_1_ (a_1_)	−0.273 *	0.234	0.006	−1.115	−0.183
X→M_2_ (a_2_)	0.376 *	0.221	<0.001	0.547	1.426
M_1_→M_2_ (a_3_)	−0.386 *	0.093	<0.001	−0.611	−0.242
M_1_→Y (b_1_)	−0.017	0.048	0.857	−0.104	0.087
M_2_→Y (b_2_)	0.503 *	0.048	<0.001	0.134	0.325
X→Y (c’)	0.115	0.113	0.229	−0.088	0.363
**Indirect Effect**					
X→M_1_→Y	0.004	0.031	-	−0.044	0.082
X→M_2_→Y	0.189 *	0.049	-	0.098	0.294
X→M_1_→M_2_→Y	0.053 *	0.025	-	0.009	0.108
**Total Effect (c)**	0.362 *	0.112	<0.001	0.210	0.657

Note. Age, sex and ADHD treatment were included as covariates. * Statistically significant (*p* < 0.05 or CIs do not include zero) S.E.: standard error; X = ADHD index; M_1_ = emotion regulation; M_2_ = Revised Children’s Anxiety and Depression Scale-Total Internalization; Y = Children’s Sleep Habits Questionnaire-Total Sleep.

**Table 7:** Results of serial mediation analysis, testing the mediator effects of emotional intensity and internalizing symptoms on the path from oppositional defiant symptoms to total sleep problems (*n* = 100).

Path	Standardized Coefficients	S.E.	*p*	95% CI	
				Lower	Upper
**Direct Effects**					
X→M_1_ (a_1_)	0.660 *	0.229	<0.001	1.529	2.442
X→M_2_ (a_2_)	0.174	0.435	0.077	−0.087	1.642
M_1_→M_2_ (a_3_)	0.583 *	0.145	<0.001	0.573	1.151
M_1_→Y (b_1_)	−0.023	0.087	0.860	−0.190	0.159
M_2_→Y (b_2_)	0.466 *	0.053	<0.001	0.107	0.318
X→Y (c’)	0.210	0.228	0.063	−0.025	0.881
**Indirect Effect**					
X→M_1_→Y	−0.015	0.070	-	−0.150	0.124
X→M_2_→Y	0.081	0.048	-	−0.011	0.181
X→M_1_→M_2_→Y	0.179 *	0.049	-	0.094	0.286
**Total Effect (c)**	0.457 *	0.184	<0.001	0.562	1.293

Note. Age, sex and ADHD treatment were included as covariates. * Statistically significant (*p* < 0.05 or CIs do not include zero). S.E.: standard error; X = Conners Parent Rating Scale-Revised Short-Oppositional Defiant Symptoms; M_1_ = emotional intensity; M_2_ = Revised Children’s Anxiety and Depression Scale-Total Internalization; Y = Children’s Sleep Habits Questionnaire-Total Sleep.

**Table 8:** Results of serial mediation analysis, testing the mediator effects of emotion regulation and internalizing symptoms on the path from oppositional defiant symptoms to total sleep problems (*n* = 100).

Path	Standardized Coefficients	S.E.	*p*	95% CI	
				Lower	Upper
**Direct Effects**					
X→M_1_ (a_1_)	−0.625 *	0.327	<0.001	−3.167	−1.866
X→M_2_ (a_2_)	0.412 *	0.474	<0.001	0.891	2.773
M_1_→M_2_ (a_3_)	−0.236 *	0.116	0.027	−0.492	−0.029
M_1_→Y (b_1_)	0.088	0.053	0.409	−0.062	0.151
M_2_→Y (b_2_)	0.475 *	0.046	<0.001	0.124	0.309
X→Y (c’)	0.246 *	0.229	0.031	0.044	0.955
**Indirect Effect**					
X→M_1_→Y	−0.055	0.075	-	−0.208	0.085
X→M_2_→Y	0.195 *	0.064	-	0.074	0.330
X→M_1_→M_2_→Y	0.070 *	0.038	-	0.005	0.159
**Total Effect (c)**	0.457 *	0.184	<0.001	0.562	1.293

Note. Age, sex and ADHD treatment were included as covariates.* Statistically significant (*p* < 0.05 or CIs do not include zero).S.E.: standard error; X = Conners Parent Rating Scale-Revised Short-Oppositional Defiant Symptoms; M_1_ = emotion regulation; M_2_ = Revised Children’s Anxiety and Depression Scale-Total Internalization; Y = Children’s Sleep Habits Questionnaire-Total Sleep.

## Data Availability

The data is available from the corresponding author upon reasonable request.

## Supplementary Materials

The following supporting information can be downloaded at: https://www.mdpi.com/article/10.3390/bs16030404/s1, Table S1. Sensitivity Analysis: Correlation Between Internalizing Symptoms and Total Sleep Problems in ADHD Children “including the sleep item” vs. “excluding the sleep item” (*n* = 100). Table S2. Comparison of Clinical and Sleep Variables by ADHD Presentation. Table S3. Sensitivity Analysis: Regression Model Conducted to Investigate Variables Associated with Total Sleep Scores (Sleep Item Excluded; ADHD Presentation Adjusted) (*n* = 100). Table S4. Sensitivity Analysis: Serial Mediation Analyses Predicting Total Sleep Problems (Sleep Item Excluded form RCADS; ADHD Presentation Adjusted) (*n* = 100).

## Abbreviations

ADHD	Attention Deficit Hyperactivity Disorder
CPRS	Conners’ Parent Rating Scale-Revised: Short Form
CSHQ	Children’s Sleep Habits Questionnaire
DSM-5	The Diagnostic and Statistical Manual of Mental Disorders-Fifth Edition
ED	Emotional Dysregulation
EI	Emotional Intensity
ER	Emotional Regulation
ERSC-A	Emotion Regulation Scale for Children-Adult Form
FMR	False Discovery Rate
K-SADS-PL	Kiddie Schedule for Affective Disorders and Schizophrenia
RCADS-P	Revised Child Anxiety and Depression Scale-Parent Form
SPSS	Statistical Package for Social Sciences
VIF	Variance Influence Factor

## References

[B1-behavsci-16-00404] Accardo J. A., Marcus C. L., Leonard M. B., Shults J., Meltzer L. J., Elia J. (2012). Associations between psychiatric comorbidities and sleep disturbances in children with attention-deficit/hyperactivity disorder. Journal of Developmental & Behavioral Pediatrics.

[B2-behavsci-16-00404] APA (2013). Diagnostic and statistical manual of mental disorders.

[B3-behavsci-16-00404] Becker S. P. (2020). ADHD and sleep: Recent advances and future directions. Current Opinion in Psychology.

[B4-behavsci-16-00404] Becker S. P., Langberg J. M., Eadeh H., Isaacson P. A., Bourchtein E. (2019). Sleep and daytime sleepiness in adolescents with and without ADHD: Differences across ratings, daily diary, and actigraphy. Journal of Child Psychology and Psychiatry.

[B5-behavsci-16-00404] Becker S. P., Langberg J. M., Evans S. W. (2015). Sleep problems predict comorbid externalizing behaviors and depression in young adolescents with attention-deficit/hyperactivity disorder. European Child & Adolescent Psychiatry.

[B6-behavsci-16-00404] Biederman J., Petty C. R., Day H., Goldin R. L., Spencer T., Faraone S. V., Surman C. B. H., Wozniak J. (2012). Severity of the aggression/anxiety-depression/attention child behavior checklist profile discriminates between different levels of deficits in emotional regulation in youth with attention-deficit hyperactivity disorder. Journal of Developmental & Behavioral Pediatrics.

[B7-behavsci-16-00404] Bondopandhyay U., McGrath J., Coogan A. N. (2024). Associations between sleep problems in children with ADHD and parental insomnia and ADHD symptoms. PLoS ONE.

[B8-behavsci-16-00404] Bunford N., Evans S. W., Langberg J. M. (2018). Emotion dysregulation is associated with social impairment among young adolescents with ADHD. Journal of Attention Disorders.

[B9-behavsci-16-00404] Chorpita B. F., Yim L., Moffitt C., Umemoto L. A., Francis S. E. (2000). Assessment of symptoms of DSM-IV anxiety and depression in children: A revised child anxiety and depression scale. Behaviour Research and Therapy.

[B10-behavsci-16-00404] Conners C. K. (1997). Conners’ rating scales-revised. Instruments for use with children Ind adolescents.

[B11-behavsci-16-00404] Corkum P., Panton R., Ironside S., MacPherson M., Williams T. (2007). Acute impact of immediate release methylphenidate administered three times a day on sleep in children with attention-deficit/hyperactivity disorder. Journal of Pediatric Psychology.

[B12-behavsci-16-00404] Cortese S., Faraone S. V., Konofal E., Lecendreux M. (2009). Sleep in children with attention-deficit/hyperactivity disorder: Meta-Analysis of subjective and objective studies. Journal of the American Academy of Child & Adolescent Psychiatry.

[B13-behavsci-16-00404] Costa-López B., Lavigne-Cerván R., Collado-Valero J. A., Juárez-Ruiz de Mier R., Navarro-Soria I. (2023). The influence of emotional regulation and cognitive flexibility on sleep habits in Spanish children and adolescents through the lens of parents. Children.

[B14-behavsci-16-00404] Déry M., Lapalme M., Jagiellowicz J., Poirier M., Temcheff C., Toupin J. (2017). Predicting depression and anxiety from oppositional defiant disorder symptoms in elementary school-age girls and boys with conduct problems. Child Psychiatry & Human Development.

[B15-behavsci-16-00404] Dimakos J., Gauthier-Gagné G., Lin L., Scholes S., Gruber R. (2021). The associations between sleep and externalizing and internalizing problems in children and adolescents with attention-deficit/hyperactivity disorder. Child and Adolescent Psychiatric Clinics of North America.

[B16-behavsci-16-00404] Dolapoglu N., Kirkan T. S., Tulaci R. G. (2025). The relationship between attention deficit hyperactivity disorder emotion regulation difficulties and sleep quality in adults: A cross sectional study. BMC Psychiatry.

[B17-behavsci-16-00404] Dong H.-Y., Miao C.-Y., Xue Y., Zhang Y., Shan L., Jia F.-Y., Du L. (2024). Sleep and internalizing problems in primary school children with attention-deficit hyperactivity disorder. Pediatric Research.

[B18-behavsci-16-00404] Dugal C., Godbout N., Bélanger C., Hébert M., Goulet M. (2018). Cumulative childhood maltreatment and subsequent psychological violence in intimate relationships: The role of emotion dysregulation. Partner Abuse.

[B19-behavsci-16-00404] Ercan E. S., Polanczyk G., Akyol Ardıc U., Yuce D., Karacetın G., Tufan A. E., Tural U., Aksu H., Aktepe E., Rodopman Arman A., Başgül S., Bılac O., Coşkun M., Celık G. G., Karakoc Demırkaya S., Dursun B. O., Durukan İ., Fidan T., Perdahlı Fiş N., Yıldız N. (2019). The prevalence of childhood psychopathology in Turkey: A cross-sectional multicenter nationwide study (EPICPAT-T). Nordic Journal of Psychiatry.

[B20-behavsci-16-00404] Esbjørn B. H., Bender P. K., Reinholdt-Dunne M. L., Munck L. A., Ollendick T. H. (2012). The development of anxiety disorders: Considering the contributions of attachment and emotion regulation. Clinical Child and Family Psychology Review.

[B21-behavsci-16-00404] Frick M. A., Meyer J., Isaksson J. (2023). The role of comorbid symptoms in perceived stress and sleep problems in adolescent ADHD. Child Psychiatry & Human Development.

[B22-behavsci-16-00404] Fulfs T., Poulain T., Vogel M., Nenoff K., Kiess W. (2024). Associations between sleep problems and emotional/behavioural difficulties in healthy children and adolescents. BMC Pediatrics.

[B23-behavsci-16-00404] Gormez V., Kilincaslan A., Ebesutani C., Orengul A. C., Kaya I., Ceri V., Nasiroglu S., Filiz M., Chorpita B. F. (2017). Psychometric properties of the parent version of the revised child anxiety and depression scale in a clinical sample of turkish children and adolescents. Child Psychiatry & Human Development.

[B24-behavsci-16-00404] Gratz K. L., Roemer L. (2004). Multidimensional assessment of emotion regulation and dysregulation: Development, factor structure, and initial validation of the difficulties in emotion regulation scale. Journal of Psychopathology and Behavioral Assessment.

[B25-behavsci-16-00404] Hayes A. (2013). Introduction to mediation, moderation, and conditional process analysis: A regression-based approach.

[B26-behavsci-16-00404] Huguet A., Izaguirre Eguren J., Miguel-Ruiz D., Vall Vallés X., Alda J. A. (2019). Deficient emotional self-regulation in children with attention deficit hyperactivity disorder: Mindfulness as a useful treatment modality. Journal of Developmental & Behavioral Pediatrics.

[B27-behavsci-16-00404] Hvolby A. (2015). Associations of sleep disturbance with ADHD: Implications for treatment. ADHD Attention Deficit and Hyperactivity Disorders.

[B28-behavsci-16-00404] Kaner S., Büyüköztürk Ş., İşeri E. (2013). Conners Anababa Dereceleme Ölçeği-Yenilenmiş Kısa: Türkiye Stardardizasyon Çalışması. Nöro Psikiyatri Arşivi.

[B29-behavsci-16-00404] Kaufman J., Birmaher B., Axelson D., Pereplitchikova F., Brent D., Ryan N. (2016). Schedule for affective disorders and schizophrenia for school-aged children: Present and lifetime version (K-SADS-PL) DSM-5, November 2016 working draft.

[B30-behavsci-16-00404] Liang X., Qiu H., Li S. X. (2023). Objectively measured sleep continuity in children and adolescents with ADHD: A systematic review and meta-analysis. Psychiatry Research.

[B31-behavsci-16-00404] Liang X., Zhao M., Su L., Haegele J. A., Xu R. H., Li J., Guo J., Tse A. C.-Y., Li S. X., Shum D. H. K. (2024). Sleep problems in children with ADHD: Associations with internalizing symptoms and physical activity. Journal of Autism and Developmental Disorders.

[B32-behavsci-16-00404] Loevaas M. E. S., Sund A. M., Patras J., Martinsen K., Hjemdal O., Neumer S.-P., Holen S., Reinfjell T. (2018). Emotion regulation and its relation to symptoms of anxiety and depression in children aged 8–12 years: Does parental gender play a differentiating role?. BMC Psychology.

[B33-behavsci-16-00404] Loram G., Ling M., Silk T., Sciberras E. (2023). Associations between ADHD, sleep problems, and mental health symptoms in adolescents. Journal of Attention Disorders.

[B34-behavsci-16-00404] Lugo-Candelas C., Flegenheimer C., McDermott J. M., Harvey E. (2017). Emotional understanding, reactivity, and regulation in young children with ADHD symptoms. Journal of Abnormal Child Psychology.

[B35-behavsci-16-00404] MacKinnon D. P., Lockwood C. M., Williams J. (2004). Confidence limits for the indirect effect: Distribution of the product and resampling methods. Multivariate Behavioral Research.

[B36-behavsci-16-00404] Marten F., Keuppens L., Baeyens D., Boyer B. E., Danckaerts M., Cortese S., Van der Oord S. (2023). Sleep parameters and problems in adolescents with and without ADHD: A systematic review and meta-analysis. JCPP Advances.

[B37-behavsci-16-00404] Mick E., Biederman J., Jetton J., Faraone S. V. (2000). Sleep disturbances associated with attention deficit hyperactivity disorder: The impact of psychiatric comorbidity and pharmacotherapy. Journal of Child and Adolescent Psychopharmacology.

[B38-behavsci-16-00404] Mindell J., Owens J. (2010). A clinical guide to pediatric sleep: Diagnosis and management of sleep problems.

[B39-behavsci-16-00404] Overgaard K. R., Oerbeck B., Aase H., Torgersen S., Reichborn-Kjennerud T., Zeiner P. (2018). Emotional lability in preschoolers with symptoms of ADHD. Journal of Attention Disorders.

[B40-behavsci-16-00404] Owens J. A. (2005). The ADHD and sleep conundrum. Journal of Developmental & Behavioral Pediatrics.

[B41-behavsci-16-00404] Owens J. A., Maxim R., Nobile C., McGuinn M., Msall M. (2000a). Parental and self-report of sleep in children with attention-deficit/hyperactivity disorder. Archives of Pediatrics & Adolescent Medicine.

[B42-behavsci-16-00404] Owens J. A., Spirito A., McGuinn M. (2000b). The children’s sleep habits questionnaire (CSHQ): Psychometric properties of a survey instrument for school-aged children. Sleep.

[B43-behavsci-16-00404] Özbaran B., Kalyoncu T., Köse S. (2018). Theory of mind and emotion regulation difficulties in children with ADHD. Psychiatry Research.

[B44-behavsci-16-00404] Paulus F. W., Ohmann S., Möhler E., Plener P., Popow C. (2021). Emotional dysregulation in children and adolescents with psychiatric disorders. A narrative review. Frontiers in Psychiatry.

[B45-behavsci-16-00404] Perdahlı Fiş N., Arman A., Topuzoglu A., Selcen GÜLER A., Gökçe İmren S., Ersu R., Berkem M. (2010). Perdahlı Fiş ve ark. 151 Çocuk Uyku Alışkanlıkları Anketinin Türkçe geçerliliği ve güvenilirliği. Anadolu Psikiyatri Dergisi.

[B46-behavsci-16-00404] Polanczyk G. V., Willcutt E. G., Salum G. A., Kieling C., Rohde L. A. (2014). ADHD prevalence estimates across three decades: An updated systematic review and meta-regression analysis. International Journal of Epidemiology.

[B47-behavsci-16-00404] Preacher K. J., Hayes A. F. (2008). Asymptotic and resampling strategies for assessing and comparing indirect effects in multiple mediator models. Behavior Research Methods.

[B48-behavsci-16-00404] Rydell A.-M., Berlin L., Bohlin G. (2003). Emotionality, emotion regulation, and adaptation among 5- to 8-year-old children. Emotion.

[B49-behavsci-16-00404] Schatz D. B., Rostain A. L. (2006). ADHD with comorbid anxiety. Journal of Attention Disorders.

[B50-behavsci-16-00404] Shanahan L., Copeland W. E., Angold A., Bondy C. L., Costello E. J. (2014). Sleep problems predict and are predicted by generalized anxiety/depression and oppositional defiant disorder. Journal of the American Academy of Child & Adolescent Psychiatry.

[B51-behavsci-16-00404] Soler-Gutiérrez A.-M., Pérez-González J.-C., Mayas J. (2023). Evidence of emotion dysregulation as a core symptom of adult ADHD: A systematic review. PLoS ONE.

[B52-behavsci-16-00404] Steiner H., Remsing L. (2007). Practice parameter for the assessment and treatment of children and adolescents with oppositional defiant disorder. Journal of the American Academy of Child & Adolescent Psychiatry.

[B53-behavsci-16-00404] Tatlı Harmancı S., Güngör Aytar F. A. (2023). Çocuklar için Duygu Düzenleme Ölçeği Çocuk Formu (ÇDDÖ) ve Yetişkin Formunun (ÇDDÖ-YF) Türkçeye Uyarlanması. Milli Eğitim.

[B54-behavsci-16-00404] Thompson R. A. (1994). Emotion regulation: A theme in search of definition. Monographs of the Society for Research in Child Development.

[B55-behavsci-16-00404] Tomic T., Mombelli S., Oana S., Ferini-Strambi L., Raballo A., Manconi M., Galbiati A., Castelnovo A. (2025). Psychopathology and NREM sleep parasomnias: A systematic review. Sleep Medicine Reviews.

[B56-behavsci-16-00404] Tonacci A., Billeci L., Calderoni S., Levantini V., Masi G., Milone A., Pisano S., Muratori P. (2019). Sympathetic arousal in children with oppositional defiant disorder and its relation to emotional dysregulation. Journal of Affective Disorders.

[B57-behavsci-16-00404] Tung I., Li J. J., Meza J. I., Jezior K. L., Kianmahd J. S. V., Hentschel P. G., O’Neil P. M., Lee S. S. (2016). Patterns of comorbidity among girls with ADHD: A meta-analysis. Pediatrics.

[B58-behavsci-16-00404] Unal F., Oktem F., Cetin Cuhadaroglu F., Cengel Kultur S. E., Akdemir D., Foto Ozdemir D., Cak H. T., Unal D., Tiras K., Aslan C., Kalayci B. M., Sultan Dogan B., Kutuk F., Yanar E., Karaokur R., Karabucak B., Karakok B., Karaer Y., Artik A. (2019). Reliability and validity of the schedule for affective disorders and schizophrenia for school-age children-present and lifetime version, DSM-5 November 2016-Turkish Adaptation (K-SADS-PL-DSM-5-T). Turkish Journal of Psychiatry.

[B59-behavsci-16-00404] Wang B., Eastwood P. R., Becker A., Isensee C., Wong J. W. Y., Huang R.-C., Runions K. C., Stewart R. M., Meyer T., Brüni L. G., Rothenberger A., Zepf F. D. (2019). Concurrent developmental course of sleep problems and emotional/behavioral problems in childhood and adolescence as reflected by the dysregulation profile. Sleep.

[B60-behavsci-16-00404] Williams K. E., Sciberras E. (2016). Sleep and self-regulation from birth to 7 years. Journal of Developmental & Behavioral Pediatrics.

