# Supplementary material for: Factors Associated with Sleep Problems in Children with ADHD: Focusing on Emotional Regulation, Emotional Intensity and Internalizing Symptoms
Source: Behav Sci (Basel). 2026 Mar 10;16(3):404. doi: 10.3390/bs16030404 (PMC13024508; doi:10.3390/bs16030404)
Supplement: Supplementary file 1 [file behavsci-16-00404-s001.zip › behavsci-4125837-supplementary.pdf]

Table S1. Sensitivity Analysis: Correlation Between Internalizing Symptoms and Total Sleep Problems in ADHD Children "including the sleep item" vs. "excluding the sleep item" (n=100)

**Variables**	**CSHQ** BR	**CSHQ** SOD	**CSHQ** SD	**CSHQ** SA	**CSHQ** NW	**CSHQ** P	**CSHQ** SDB	**CSHQ** DS	**CSHQ** Total
**RCADS-Internalization (with sleep items)**	.386*	.140	.155	.404*	.270*	.351*	.328*	.316*	.538*
**RCADS-Internalization (with no sleep items)**	.371*	.130	.137	.400*	.267*	.347*	.322*	.302*	.520*

CSHQ: Children's Sleep Habits Questionnaire; BR: Bedtime resistance; SOD: Sleep onset delay; SD: Sleep duration; SA: Sleep anxiety; NW: Night wakings; P: Parasomnias; SDB: Sleep disordered breathing; DS: Daytime sleepiness; RCADS: Revised Children's Anxiety and Depression Scale

Table S2. Comparison of Clinical and Sleep Variables by ADHD Presentation

Variables	ADHD-Predominantly Inattention (n=33) mean ± SD	ADHD Combined/ Predominantly Hyperactive (n=67) mean ± SD	Statistical Analysis		
			t	p	Effect size
**RCADS-Internalization (with no sleep items)**	24.12 ± 19.27	31.91 ± 17.72	-2.01	.047	0.42
**Emotional Intensity**	43.30 ± 13.60	55.30 ± 10.56	-4.85	< .001	1.05
**Emotion Regulation**	91.39 ± 19.39	77.87 ± 14.26	3.95	< .001	0.86
**CSHQ-Total**	44.61 ± 6.83	49.45 ± 9.12	-2.70	.008	0.58

CSHQ: Children's Sleep Habits Questionnaire; RCADS: Revised Children's Anxiety and Depression Scale

Table S3. Sensitivity Analysis: Regression Model Conducted to Investigate Variables Associated with Total Sleep Scores (Sleep Item Excluded; ADHD Presentation Adjusted) (n=100)

**Variables (DV: CSHQ-Total)**	**B**	**SE**	**β**	**p**
Age	-.498	.335	-.132	.140
ADHD Index	.025	.136	.021	.851
Oppositional Defiance	.514	.300	.253	.090
EI	.009	.093	.013	.923
ER	.040	.058	.080	.488
RCADS Internalization (with no sleep items)	.192	.058	.409	.001*
**ADHD Presentation**	.521	1.945	.028	.789

P values marked with an asterisk* are statistically significant. *R^2 = .347, Adjusted R^2 = .298, F(7, 92) = 6.99, p < .001*
DV: Dependent Variable; EI: Emotional Intensity; ER: Emotion Regulation; RCADS: Revised Children's Anxiety and Depression Scale

Table S4. Sensitivity Analysis: Serial Mediation Analyses Predicting Total Sleep Problems (Sleep Item Excluded form RCADS; ADHD Presentation Adjusted) (n=100)

**Model**	**Direct effect (c′)**	**Total indirect effect**	**Ind1: X→M1→Y (95% CI)**	**Ind2: X→M2→Y (95% CI)**	**Ind3: X→M1→M2→Y (95% CI)**
ADHD Index → EI → RCADS → Sleep	.091 (p = .466)	.283 [.134, .475]	.019 [-.071, .131]	.138 [.058, .237]	.126 [.046, .234]
ADHD Index → ER → RCADS → Sleep	.094 (p = .449)	.280 [.145, .462]	-.001 [-.044, .073]	.244 [.119, .406]	.036 [-.013, .096]
Oppositional → EI → RCADS → Sleep	.404 (p = .116)	.516 [.217, .871]	-.028 [-.293, .253]	.202 [.020, .419]	.342 [.155, .579]
Oppositional → ER → RCADS → Sleep	.473 (p = .072)	.448 [.104, .768]	-.107 [-.457, .163]	.419 [.167, .749]	.136 [.011, .306]

Note. Age, sex, ADHD treatment and **ADHD presentation** were included as covariates.
Indirect effects are considered statistically significant when the 95% bootstrap confidence interval does not include zero.
EI = Emotional Intensity; ER = Emotion Regulation; Internalizing = RCADS internalizing score **excluding sleep item.**
